# Meta-Analysis of In Vitro Antimicrobial Capacity of Extracts and Essential Oils of *Syzygium aromaticum*, *Citrus* L. and *Origanum* L.: Contrasting the Results of Different Antimicrobial Susceptibility Methods

**DOI:** 10.3390/foods12061265

**Published:** 2023-03-16

**Authors:** Beatriz Nunes Silva, Olga María Bonilla-Luque, Arícia Possas, Youssef Ezzaky, Abdelkhaleq Elmoslih, José António Teixeira, Fouad Achemchem, Antonio Valero, Vasco Cadavez, Ursula Gonzales-Barron

**Affiliations:** 1 Centro de Investigação de Montanha (CIMO), Instituto Politécnico de Bragança, Campus de Santa Apolónia, 5300-253 Bragança, Portugal; beatrizsilva@ceb.uminho.pt (B.N.S.);; 2 Laboratório para a Sustentabilidade e Tecnologia em Regiões de Montanha, Instituto Politécnico de Bragança, Campus de Santa Apolónia, 5300-253 Bragança, Portugal; 3 CEB—Centre of Biological Engineering, University of Minho, Campus de Gualtar, 4710-057 Braga, Portugal; 4 LABBELS—Associate Laboratory, 4710-057 Braga, Portugal; 5 Departamento de Bromatología y Tecnología de los Alimentos, UIC Zoonosis y Enfermedades Emergentes ENZOEM, ceiA3, Campus Rabanales, Universidad de Córdoba, 14014 Córdoba, Spain; 6 Bioprocess and Environment Team, LASIME Lab., Agadir Superior School of Technology, Ibn Zohr University, Agadir 80150, Morocco

**Keywords:** foodborne pathogens, inhibition diameter, minimum inhibitory concentration, meta-regression, mixed-effects model

## Abstract

Diffusion methods, including agar disk-diffusion and agar well-diffusion, as well as dilution methods such as broth and agar dilution, are frequently employed to evaluate the antimicrobial capacity of extracts and essential oils (EOs) derived from *Origanum* L., *Syzygium aromaticum*, and *Citrus* L. The results are reported as inhibition diameters (IDs) and minimum inhibitory concentrations (MICs), respectively. In order to investigate potential sources of variability in antimicrobial susceptibility testing results and to assess whether a correlation exists between ID and MIC measurements, meta-analytical regression models were built using in vitro data obtained through a systematic literature search. The pooled ID models revealed varied bacterial susceptibilities to the extracts and in some cases, the plant species and methodology utilised impacted the measurements obtained (*p* < 0.05). Lemon and orange extracts were found to be most effective against *E. coli* (24.4 ± 1.21 and 16.5 ± 0.84 mm, respectively), while oregano extracts exhibited the highest level of effectiveness against *B. cereus* (22.3 ± 1.73 mm). Clove extracts were observed to be most effective against *B. cereus* and demonstrated the general trend that the well-diffusion method tends to produce higher ID (20.5 ± 1.36 mm) than the disk-diffusion method (16.3 ± 1.40 mm). Although the plant species had an impact on MIC, there is no evidence to suggest that the methodology employed had an effect on MIC (*p* > 0.05). The ID–MIC model revealed an inverse correlation (R^2^ = 47.7%) and highlighted the fact that the extract dose highly modulated the relationship (*p* < 0.0001). The findings of this study encourage the use of extracts and EOs derived from *Origanum*, *Syzygium aromaticum*, and *Citrus* to prevent bacterial growth. Additionally, this study underscores several variables that can impact ID and MIC measurements and expose the correlation between the two types of results.

## 1. Introduction

Plant extracts and essential oils (EOs) have potential as antimicrobial agents, owing to their rich secondary metabolites (e.g., phenols, terpenoids, and alkaloids) [1]. Several studies have investigated the in vitro antimicrobial activity of *Origanum* L., *Syzygium aromaticum*, and *Citrus* L. extracts and EOs against foodborne pathogens, yielding encouraging results [2,3,4,5,6,7].

A range of in vitro assays can be utilised to determine the susceptibility of a microorganism to antimicrobial agents, including diffusion methods (agar disk-diffusion and agar well-diffusion) and dilution methods (broth and agar dilution), with standardised methods available from CLSI, ISO, and EUCAST [8,9,10,11]. The agar disk-diffusion method involves placing paper disks containing the test compound on a bacterial lawn on the surface of an agar medium at a specific concentration, while the agar well-diffusion method involves placing a pre-defined volume of the antimicrobial agent at a specific concentration into a hole of 6 to 8 mm in diameter punched aseptically into the agar [12]. Both methods require incubation under suitable conditions, followed by measurement of the diameters of inhibition zones around the disks or wells [12]. However, it is important to note that these diffusion methods have some limitations, including the inability to differentiate between bactericidal and bacteriostatic effects and to establish the minimum inhibitory concentration (MIC), due to the difficulty in calculating the quantity of the antimicrobial agent that has diffused into the agar medium [12].

Alternatively, dilution methods, unlike diffusion methods, are well-suited for determining MIC values, as they allow for estimation of the antimicrobial concentration in both broth (macro-dilution or micro-dilution) and agar medium (agar dilution) [12]. In the agar dilution method, the antimicrobial agent is incorporated into liquid agar medium at varying concentrations, followed by inoculation of a standardised bacterial inoculum onto the agar plate surface [12,13]. Broth macro- and micro-dilution methods involve placing a standardised bacterial suspension into tubes (macro) or 96-well trays (micro) filled with a liquid medium of predetermined formulation and two-fold serial dilutions of the antimicrobial agent to be tested [12,13]. After adequate incubation of agar plates, tubes, or trays, MIC values are determined through visual or spectrophotometric inspection, depending on the protocol employed [8,9,10,11,12].

With diffusion and dilution methods reporting antimicrobial activity in terms of inhibition diameter (in millimetres) and MIC (in mg/mL, for example) of the bacterium being tested, respectively, the following question was raised: can a relationship be detected between inhibition diameter and MIC values obtained from different in vitro methodologies? Moreover, how are the results affected by the method used (disk- vs. well-diffusion; broth vs. agar dilution)? To investigate and answer these questions, a meta-analysis was conducted on the antibacterial capacity of *Syzygium aromaticum*, *Citrus*, and *Origanum* species extracts and EOs. While some studies have attempted to compare and correlate results obtained by different methods [14,15,16,17,18], to the best of our knowledge, this is the first time that a meta-analysis has been used to investigate the relationship between inhibition zone diameters and MIC and quantify the heterogeneity among antimicrobial susceptibility tests. Meta-analysis is a statistical synthesis technique that combines the results from various studies to produce a more precise and statistically powerful estimate of the effect of a specific treatment [19]. Furthermore, it allows identification and quantification of heterogeneity sources between the outcomes of the studies [19].

Our study aims to use systematic literature search and meta-regression modelling to achieve the following goals: (i) to collate and summarise publicly accessible data on the antimicrobial properties of *Citrus*, *Origanum*, and *Syzygium aromaticum* extracts and EOs in vitro; (ii) to examine the presence of heterogeneity in the observed effect sizes of antimicrobial activity and, if present, to identify its sources using multilevel meta-analyses and coded study characteristics; (iii) to investigate whether a relationship exists between inhibition diameter and MIC values obtained from different in vitro procedures; and (iv) to evaluate likelihood of publication bias, which is defined as “the failure to publish the study results based on the direction or strength of the study’s findings” [20].

## 2. Materials and Methods

### 2.1. Collection and Characterisation of the Dataset

A rigorous electronic search of the Web of Science, PubMed, Scopus, and SciELO databases was performed to identify high-quality, peer-reviewed, original publications since 2000 which reported data on inhibition diameter, MIC, and minimum bactericidal concentration (MBC) of extracts derived from *Origanum*, *Syzygium*, and *Citrus*. The aim of the search was to locate studies that had been validated by the scientific community.

The logical connectors “and” and “or” were appropriately utilised to merge terms related to biopreservatives, pathogens, and antimicrobial susceptibility testing methodologies in the electronic search. The following terms were used: (*Listeria* or *Salmonella* or “*Staphylococcus aureus*” or “*Escherichia coli*” or *Campylobacter*) and (extract* or antimicrobial* or “essential oil”) and (MIC or MBC or “agar diffusion” or halo or inhibition or zone or “minimum inhibitory concentration” or “minimum bactericidal concentration”) and food. The search was conducted in the title, keywords, and abstract to identify high-quality studies validated by the scientific community and covered articles published from 2000 onwards.

The study excluded grey literature, meta-analyses, and systematic reviews to avoid data duplication and ensure data validity. The inclusion criteria specified *Origanum*, *Syzygium*, or *Citrus* extracts or EOs with either MIC or inhibition diameter measurements against selected foodborne pathogens, including Shiga toxin-producing *E. coli* (STEC), *S. aureus*, *L. monocytogenes*, *Salmonella* spp., and *Campylobacter* spp. The extract dosage and pathogen inoculum size were also required. The selected bacteria were chosen for their frequent use in antimicrobial susceptibility testing and their importance as causative agents of foodborne diseases [21].

After evaluating the collected publications, a total of 131 papers published since 2000 were considered appropriate for inclusion [2,4,5,6,7,22,23,24,25,26,27,28,29,30,31,32,33,34,35,36,37,38,39,40,41,42,43,44,45,46,47,48,49,50,51,52,53,54,55,56,57,58,59,60,61,62,63,64,65,66,67,68,69,70,71,72,73,74,75,76,77,78,79,80,81,82,83,84,85,86,87,88,89,90,91,92,93,94,95,96,97,98,99,100,101,102,103,104,105,106,107,108,109,110,111,112,113,114,115,116,117,118,119,120,121,122,123,124,125,126,127,128,129,130,131,132,133,134,135,136,137,138,139,140,141,142,143,144,145,146,147,148,149,150]. The information collected from the chosen studies includes article identification, plant species, plant portion used, extraction method including its parameters such as temperature and solvent, antimicrobial susceptibility test, extract or EO dosage applied (“LogDose”; %*w*/*v* or %*v*/*v*), bacterium, strain, inoculum size, inhibition diameter value (ID, mm), and MIC value (“LogMIC”; mg/mL for extracts, μL/mL for EOs).

### 2.2. Meta-Regression Modelling

Weighted mixed-effects linear models were utilised to estimate pooled inhibition diameters or MIC values produced by extracts or EOs of *Syzygium aromaticum*, *Origanum*, and *Citrus* species against specific bacteria. For each dataset, study characteristics were extracted from primary studies to explain variability in effect size between studies. These characteristics included plant type, extract or EO dose tested, volume of extract or EO absorbed or poured, inoculum level, method of determining inhibition diameter, and number of replicates used for test. Pooled MIC models were codified based on plant type, method of determination of MIC/MBC, standard errors, antimicrobial type (extract or EO), and number of replicates used for the test. Interactions between factors were evaluated in some models to determine if the effect of one term depended on the level of one or more terms. Over 30 meta-regression models were adjusted to synthesise inhibition diameter (ID) and MIC, using a general form (Equations (1) and (2)):

(1) $$ID_{ij}=\beta_{1}LogDose+\left(\beta_{2j}+u_{i}\right)Plant_{j}+\varepsilon_{ij}$$

(2) $$logMIC_{ijmn}=\left(\beta_{1j}+u_{i}\right)Plant_{j}+\beta_{2m}Method_{m}+\beta_{3n}AntimicrobialType_{n}+\varepsilon_{ijmn}$$

Equation (1) provides the model used to estimate the ID, where $ID_{ij}$ refers to the ID observation obtained from the *j*-th plant and the *i*-th study. The effect of a one log increase in extract dose (%*v*/*v* or %*w*/*v*) on the inhibition diameter is represented by $\beta_{1}$. Additionally, the fixed effects of the *j* types of plant are captured by $\beta_{2j}$.

Similarly, Equation (2) represents the model used to estimate the MIC produced by plant extracts, where $MIC_{ijmn}$ refers to the MIC observation obtained from the *j*-th plant, the *m*-th method of MIC determination (which can be agar dilution or broth micro-dilution), the *n*-th antimicrobial type (extract or EO), and the *i*-th study. The fixed effects of the *j* categories of plant, *m* types of MIC determination method, and *n* types of antimicrobial test are represented by $\beta_{1j}$, $\beta_{2m}$, and $\beta_{3n}$, respectively.

The terms $\varepsilon_{ij}$ and $\varepsilon_{ijmn}$ of Equations (1) and (2), respectively, are the model residuals. The remaining unexplained variability was extracted by introducing random effects $u_{i}$ due to study *i* in $\beta_{2j}$ and $\beta_{1j}$ (set of fixed effects of the *j* types of plant in Equations (1) and (2), respectively). In both models, the terms $u_{i}$ are assumed to follow a normal distribution with mean zero and between-study variability $\tau^{2}$.

The correlation between inhibition diameter and MIC of different pathogens produced by extracts or EOs of *Syzygium aromaticum*, *Origanum*, and *Citrus* species was examined by adjusting another weighted mixed-effects linear model to the corresponding dataset. The moderators considered in this model included the logarithm of the extract dose, logarithm of the MIC, and bacterium. The adjusted meta-regression model had the following form:

(3) $$ID_{ik}=\left(\beta_{0}+u_{i}\right)+\beta_{1}LogDose+\beta_{2}LogMIC+\beta_{3k}Bacterium_{k}+\varepsilon_{ik}$$

Equation (3) specifies the model adjusted, where $\beta_{0}$ is an intercept and $\beta_{1}$ and $\beta_{2}$ represent the effect of a one log increase in extract dosage (%*v*/*v* or %*w*/*v*) and of a one log increase in MIC (mg/mL for extracts and μL/mL for EO), respectively, on the inhibition diameter. The set of fixed effects of the *k* bacteria types is denoted by $\beta_{3k}$. The error term $\varepsilon_{ik}$ accounts for the variability between pathogens *k* and studies *i*. The remaining unexplained variability was extracted by placing random effects $u_{i}$ due to study *i* in $\beta_{0}.$

All models were adjusted by logarithmically transforming (base-10) the extract or EO dose tested, as well as MIC values, to normalise data distribution and reduce heteroscedasticity. Moreover, weights were allocated to each primary study based on its sample size, *n* (*n* ≥ 2), with the aim of capturing the quality of research design and obtaining accurate estimations of the antimicrobial effect on pathogen inactivation.

The model parameters, influenced by moderators, were derived from the fitted meta-regressions and assessed for significance through analysis of variance (ANOVA, α = 0.05). Two methods were employed to evaluate publication bias: (1) analysis of funnel plot and (2) examination of the effect of the total sample size of the study (n) on the pooled ID/MIC [19,151]. The meta-regression models were fitted using the metafor package available in R software (version 4.1.0, R Foundation for Statistical Computing, Vienna, Austria) [152], in particular the *rma.mv* function.

## 3. Results and Discussion

It is noteworthy that the synthesised results of inhibition diameters and MIC of *Citrus*, *Origanum*, and *Syzygium aromaticum* species against specific pathogens form the basis of this meta-analysis. As such, the estimates presented herein cannot be extrapolated to different plant species or bacteria.

### 3.1. Inhibition Diameter

#### 3.1.1. *Citrus* Species

The inhibition diameters produced by EOs of *Citrus* species were pooled, and resulting estimates are presented in Table 1. The meta-analysis models were separately adjusted for four specific pathogens, namely *E. coli*, *S. aureus*, *Salmonella*, and *L. monocytogenes*. The inhibition diameters collected from primary studies and used in the meta-analysis models were determined using the disk-diffusion method. Thus, the influence of the method of determination could not be assessed.

Considering only the outcomes pertaining to lemon and orange EOs, since they were observed across all meta-analysis models, *E. coli* was found to be the most susceptible bacterium (*p* < 0.05), whereas *S. aureus*, *Salmonella*, and *L. monocytogenes* exhibited similar levels of reduced susceptibility.

The inhibitory effect of *Citrus* EOs against *S. aureus* and *L. monocytogenes* was not significantly different (*p* > 0.05) among the investigated species, as indicated by the equal superscript lowercase letters for both models. In contrast, the effect on *Salmonella* and *E. coli* varied (*p* < 0.05) depending on the EO tested. *E. coli* exhibited similar inhibition caused by the EOs of lemon, lime, mandarin, and *Citrus* hybrids but lower inhibition when exposed to orange EO.

Publication bias was evaluated by introducing the total sample size of a study (n) as a moderator in the multilevel meta-analysis. If the effect of sample size is significant, it suggests that non-significant studies may not have been published, indicating the existence of publication bias. Of the meta-analysis models examined, only the one adjusted for *S. aureus* suggests the possibility of publication bias (*p* = 0.002).

However, since some studies do not report sample size, the presence of publication bias can also be evaluated through funnel plots. This method may be inconclusive as it relies on visual inspection rather than statistical significance. In a funnel plot, if there is no publication bias, larger studies (with larger sample sizes) will cluster around the average, while smaller studies will be evenly distributed on both sides of the average, resulting in a funnel-shaped distribution of data points. Any deviation from this pattern or the presence of large gaps may suggest publication bias, though these deviations may also be due to other factors, such as study heterogeneity. The funnel plots of these meta-analysis models are presented in Figure S1 in the Supplementary Materials.

#### 3.1.2. *Origanum* Species

Table 2 displays the results of meta-analysis models that estimated the pooled inhibition diameters produced by *Origanum* species extracts against *E. coli*, *B. cereus*, *S. aureus*, *Salmonella*, *L. monocytogenes*, and STEC.

Based on the pooled inhibition diameters presented in Table 2, it was observed that *E. coli* was the least susceptible bacterium to oregano extracts at a concentration of 100 mg/mL (*p* < 0.05), while the remaining bacteria showed comparable levels of susceptibility, namely *S. aureus*, *Salmonella*, *L. monocytogenes*, and STEC (in no particular order). The antimicrobial action of *Origanum* extracts was found to be influenced by the plant species for most bacteria, as indicated by the different superscript lowercase letters in Table 2. For instance, the extracts of marjoram, oregano, and “others” (which includes *O. dictamnus*, *O. syriacum*, and *O. minutiflorum*) differently (*p* < 0.05) inhibited the growth of *S. aureus* and *L. monocytogenes*. However, no significant difference (*p* > 0.05) was observed in the case of *E. coli*, as it was equally (*p* > 0.05) affected by marjoram and oregano extracts.

The impact of the method used to determine the inhibitory activity of oregano extracts against all bacteria was evaluated in the adjusted models, as observations were available for two distinct methods, disk- and well-diffusion. Only in the model adjusted for *L. monocytogenes* were differences (*p* < 0.05) observed between the methods. Specifically, the well method produced a superior pooled inhibition diameter (21.49 ± 1.015 mm) compared to the disk method (18.66 ± 1.877 mm). However, it should be noted that in the remaining models, a non-significant effect (*p* > 0.10) of the technique was detected. Consequently, the inhibition diameters from both the disk and well methods were merged and denoted as “Disk and Well”. Moreover, it is noteworthy that none of the models generated for *Origanum* species revealed any signs of publication bias (*p* > 0.05). A graphical depiction of the funnel plots of these models is presented in Figure S2 of the Supplementary Materials.

#### 3.1.3. *Syzygium aromaticum*

Table 3 displays the pooled inhibition diameters obtained by extracts of *Syzygium aromaticum* (clove), as estimated by meta-analysis models separately adjusted for six bacterial strains: *E. coli*, *B. cereus*, *S. aureus*, *Salmonella*, *L. monocytogenes*, and STEC.

According to the pooled inhibition diameters obtained, *B. cereus* exhibited the highest susceptibility to clove extracts at a concentration of 100 mg/mL, followed by *E. coli* and *S. aureus*. On the other hand, *Salmonella*, *L. monocytogenes*, and STEC were found to be the least susceptible to the antimicrobial effects of clove extracts.

The effect of determination method on the pooled inhibition diameters was evaluated for all bacteria, except STEC, as observations using both disk- and well-diffusion methods were available. Significant differences (*p* < 0.05) between the methods were observed in models adjusted for *E. coli*, *B. cereus*, and *S. aureus*. In all models, the well method produced higher pooled inhibition diameters (*E. coli* = 18.08 ± 1.123 mm; *B. cereus* = 20.53 ± 1.359 mm; *S. aureus* = 20.10 ± 2.613 mm) than the disk method (*E. coli* = 14.60 ± 0.894 mm; *B. cereus* = 16.29 ± 1.399 mm; *S. aureus* = 12.86 ± 1.032 mm). However, the effect of the determination method was not significant (*p* > 0.10) in the models adjusted for *Salmonella* and *L. monocytogenes*.

Three of the models produced indicated the presence of publication bias: those adjusted for *B. cereus* (*p* = 0.044), *L. monocytogenes* (*p* = 0.042), and STEC (*p* = 0.004). The funnel plots of all models are presented in Figure S3 of the Supplementary Materials.

### 3.2. Minimum Inhibitory Concentration

#### 3.2.1. *Citrus* Species

The pooled MICs produced by extracts or EOs of *Citrus* species, as estimated by meta-analysis models separately adjusted for *E. coli*, *B. cereus*, *S. aureus*, *Salmonella*, and *L. monocytogenes*, are presented in Table 4.

Significant differences (*p* < 0.05) were observed in MIC values of extracts or EOs of different *Citrus* species for all bacteria except *B. cereus*, as evidenced by the distinct superscript lowercase letters in Table 4. The hybrids category displayed the lowest MIC in models adjusted for *E. coli*, *S. aureus*, *Salmonella*, and *L. monocytogenes*. However, it is important to note that the hybrids category is a group of various *Citrus* species, and lower MIC does not necessarily imply greater efficacy against the mentioned pathogens compared to other species such as bitter orange or lime. Nonetheless, it does suggest that the plant species reported in literature that comprise the hybrids category generally possess greater antimicrobial potency than other species, including bitter orange or lime.

The effect of determination method was evaluated for orange extracts in the models adjusted for *E. coli* and *S. aureus*, and no differences (*p* > 0.05) were found in pooled MIC values obtained using either agar dilution or broth micro-dilution. Furthermore, a comparison of the pooled MIC values between EO and extracts was conducted for *Citrus* hybrids against *L. monocytogenes*, and no significant differences (*p* > 0.05) were observed between the outcomes, suggesting that these extracts and EO possess comparable antimicrobial effect.

None of the models produced indicate the presence of publication bias (*p* > 0.05). Figure S4 in the Supplementary Materials displays the funnel plots of these models.

#### 3.2.2. *Origanum* Species

The pooled MICs produced by extracts or EOs of *Origanum* species, as estimated by meta-analysis models separately adjusted for *E. coli*, *B. cereus*, *S. aureus*, *Salmonella*, *L. monocytogenes*, and STEC, are presented in Table 5.

In some cases, the extracts or EOs derived from distinct *Origanum* species were found to have a significant impact (*p* < 0.05) on the pooled MIC values of *E. coli*, *S. aureus*, *Salmonella*, and *L. monocytogenes*, as indicated by the varying superscript lowercase letters in Table 5. However, in the case of *B. cereus* and STEC models, the effect of plant species could not be evaluated as observations were limited to oregano species exclusively.

In general, oregano extracts and EOs exhibited greater antimicrobial activity than extracts from other plant species, such as marjoram. However, differences (*p* < 0.05) in inhibitory activity were observed between extracts and EOs originating from the same plant species but only in some of the models (those adjusted for *Salmonella* and *L. monocytogenes*). Moreover, the method of MIC determination significantly affected the results for oregano extracts and EOs in models adjusted for *S. aureus* and *L. monocytogenes.* For the *E. coli* model, agar dilution, broth macro-dilution, and broth micro-dilution yielded similar MIC values for oregano extracts (*p* > 0.10).

Publication bias was not detected (*p* > 0.05) in any of the models, except for the one adjusted for *B. cereus* (*p* = 0.021). A graphical representation of these outcomes is shown in Figure S5 of the Supplementary Materials.

#### 3.2.3. *Syzygium aromaticum*

The pooled MICs produced by extracts or EOs of clove, as estimated by meta-analysis models separately adjusted for *E. coli*, *B. cereus*, *S. aureus*, *Salmonella*, and *L. monocytogenes*, are presented in Table 6.

### 3.3. Inhibition Diameter as a Function of MIC, Extract Dose, and Bacterium

Table 7 presents the parameters estimated from the meta-regression model that capture the relationship between the inhibition diameter generated by extracts of *Origanum*, *Syzygium aromaticum* and *Citrus* and the MIC, extract dose, and bacterium.

**Figure 1:**
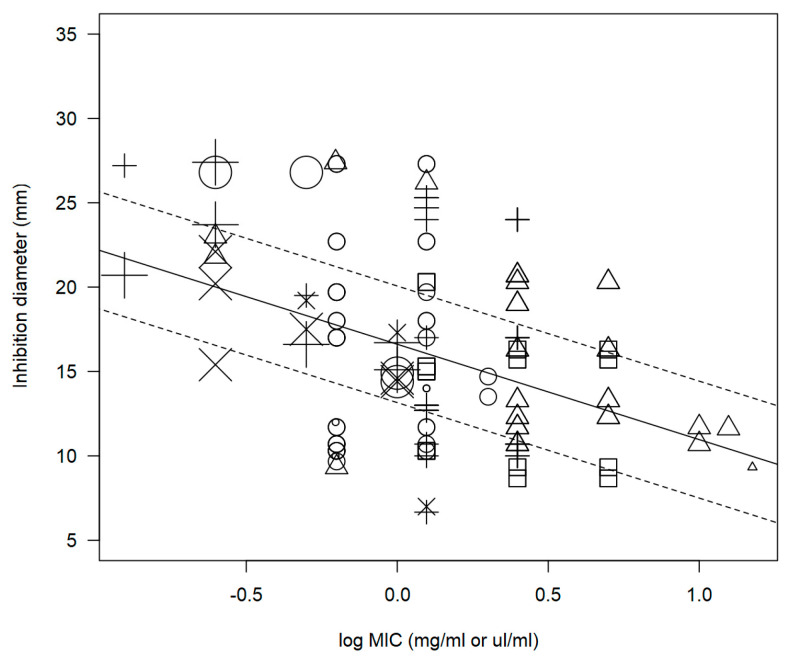
Scatter plot depicting the effect of the logarithm of the MIC (log MIC) of *Origanum* (*n* = 145), *Syzygium aromaticum* (*n* = 10), and *Citrus* (*n* = 7) extracts on inhibition diameters for each bacterium. Markers symbolise the following: □ = *C. jejuni*, ○ = *L. monocytogenes*, ∆ = *S. aureus*, + = *Salmonella*, × = STEC. The size of each marker corresponds to the sample size, with larger markers representing larger study populations.

The impact of certain moderating factors on the association between inhibition diameter and MIC was evaluated. Overall, the results of the statistical analysis indicated an inclination towards an inverse correlation, as demonstrated by the negative intercept (−1.515 ± 6.499). Notably, the negative estimate of “Log MIC” (−5.554 ± 0.181, *p* < *0*.0001) suggested an inverse correlation between this moderator and inhibition diameter. Specifically, a higher MIC was associated with a reduced efficacy of the plant extract in suppressing microbial growth. Consequently, the testing of such plant extract at the given concentration via any diffusion or dilution method resulted in a smaller diameter of inhibition. Despite the influence of various factors affecting the measurements, this relationship persisted, as exemplified by the negative slope illustrated in the scatter plot depicted in Figure 1.

Conversely, the positive estimate of “Log dose” (18.00 ± 0.227, *p* < 0.0001) suggests that there is a tendency for the inhibition diameter to increase as the dosage of the extract applied increases.

Table 7 demonstrates that different pathogens exhibit distinct inhibition diameters when subjected to the same plant extract at the same dose, as indicated by the various estimates of the moderating variable “Bacterium”. In this model, the estimate for *Campylobacter jejuni* served as the base value for inhibition diameter, with a mean of zero, and the estimates for the remaining microorganisms represent deviations from this mean, with positive and negative estimates above and below the base value, respectively. Based on these findings, *S. aureus* demonstrated the most substantial deviation in inhibition diameter when exposed to a specific plant extract at a certain dose (2.668 ± 0.146), followed by *Salmonella* (2.429 ± 0.141) and *L. monocytogenes* (1.319 ± 0.150). In contrast, STEC was the most resilient pathogen to the action of such antimicrobial agents, as indicated by the least deviation in inhibition diameter (−0.411 ± 0.234). Notably, no significant differences (*p* < 0.05) were detected between the inhibition diameters estimated for *S. aureus* and *Salmonella*, although these differed from the remaining pathogens. However, no discernible difference between the effects of the extract on Gram-positive and Gram-negative bacteria was observed in the meta-analytical models produced for the pooled inhibition diameters (Table 1, Table 2 and Table 3). This finding is consistent with the conclusions of other researchers who have reported no differences between the two types of bacteria [153], despite theoretical differences in cell wall structure, composition, and other mechanisms [154].

Upon analysis of the model produced and in conjunction with Figure S7 of the Supplementary Materials, no evidence of publication bias (*p* = 0.254) was detected.

The measurement of heterogeneity in the inhibition diameter can be quantified by the intra-class correlation, I^2^, which represents the proportion of total variability that arises from differences between studies. For this, an I^2^ value of 53.6% indicates that over half of the total variability observed in effect sizes is due to genuine heterogeneity between studies rather than mere sampling error. This level of heterogeneity is classified as medium according to Higgins and Thompson, who consider an I^2^ value around 25% or 75% to indicate low and high heterogeneity, respectively [155]. Additionally, a heterogeneity analysis was conducted to determine the extent to which moderators incorporated into the meta-regression model can explain between-study variability. The results indicate that the moderators accounted for 47.7% of the variability between studies (R^2^), leaving some residual variability unaccounted for by the model. Potential sources of variation that may explain the residual variability include factors such as the origin of the plant extract, the developmental stage and plant part used, as well as the inoculum size and strain employed. The inclusion of these factors in the models would be expected to increase the percentage of variability that can be explained. This R^2^ value also suggests that disk diffusion methodologies may not be appropriate to compare results from different studies, as the inhibition diameter measurements could be affected by errors and variations in the protocols, impacting on the degree of extract diffusion within the agar matrix.

**Figure 2:**
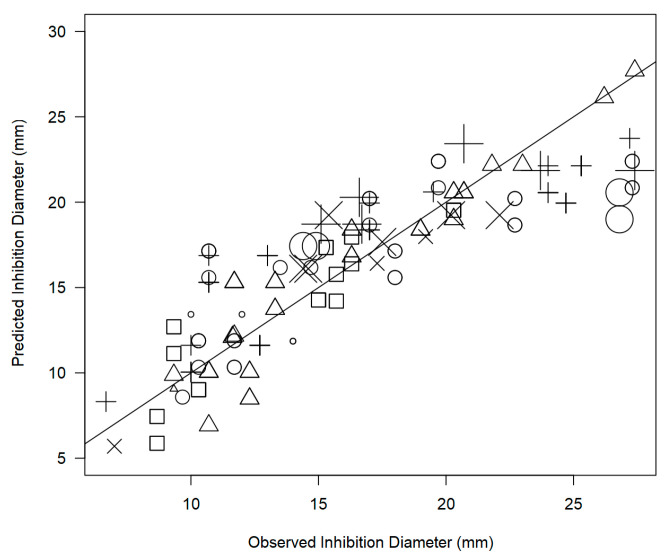
Scatter plot depicting inhibitory effects of extracts derived from *Origanum* (*n* = 145), *Syzygium aromaticum* (*n* = 10), and *Citrus* (*n* = 7) plants against predicted values generated by the meta-regression model (R^2^ = 0.860), with 45° reference line. Symbols represent different bacterial strains: □ = *C. jejuni*, ○ = *L. monocytogenes*, ∆ = *S. aureus*, + = *Salmonella*, × = STEC; marker size corresponds to the sample size of the respective study.

The evaluation of the model’s goodness of fit was conducted by plotting the predicted inhibition diameter against the observed, as depicted in Figure 2. The resulting correlation coefficient value (R^2^ = 0.860) is deemed satisfactory for a meta-analysis study, indicating a robust fundamental relationship between the two antimicrobial susceptibility determinations.

While the developed model may not account for all sources of variability within the literature, its results are nevertheless valuable as they offer valuable insight into the comparative effectiveness of extracts and EOs derived from *Syzygium aromaticum*, *Origanum*, and *Citrus* species against various organisms as well as the effect of dosage on biopreservatives’ efficacy. Such findings have practical applications in selecting suitable pathogen control measures for use in food products or packaging, aligning with current trends in the food industry that emphasise the development of novel preservatives.

## 4. Conclusions

Meta-regression analyses of pooled inhibition diameters demonstrated varied bacterial susceptibilities, with some instances of the plant species and methodology used (disk- vs. well-diffusion) having an impact. Of note, *E. coli* displayed the highest sensitivity to *Citrus* EOs, while extracts from *Origanum* and *S. aromaticum* were most effective against *B. cereus*. In situations where these pathogens are a particular concern in a given food product, the addition of such antimicrobial agents could be suggested to provide an inhibitory effect, thereby enhancing food safety. Models for pooled MIC generally revealed no effect of the methodology used (agar, broth micro- or macro-dilution) or differences between the antimicrobial capacity of extracts compared to EOs. However, some exceptions were observed. For *Citrus* and *Origanum*, the plant species had an impact on MIC values. The model for inhibition diameter as a function of MIC demonstrated an inverse correlation between the two variables while also summarising the reduction in various pathogen populations and elucidating the inhibitory capacity by extract dose. It further revealed that numerous aspects may affect the measurements of inhibition diameter, and thus comparison of results from different studies using the disk-diffusion method must be conducted carefully. While meta-analysis is not without limitations, the outcomes of these models support the potential of *Origanum*, *Syzygium aromaticum*, *Citrus* extracts, and essential oils to hinder or decelerate bacterial growth. Additionally, they provide insight into the variables affecting inhibition diameter and MIC measurements.

## Figures and Tables

**Table 1:** Pooled inhibition diameters (mean and standard error, SE, in mm) of *Citrus* species EOs against specific bacteria using separate meta-analysis models. Number of observations (n), number of primary studies (N), and *p*-value of the publication bias test are presented for each model.

Bacterium ^1^	Plant	Pooled Inhibition Diameter ^2^ (SE) (mm)	n	N	Pub. Bias (*p*-Value)
*E. coli* ^A^	Hybrids ^3^	23.68 ^a^ (2.320)	13		
	Lemon	24.43 ^a^ (1.205)	43		
	Lime	18.76 ^a^ (0.971)	11	20	0.402
	Mandarin	19.11 ^a^ (1.392)	9		
	Orange	16.48 ^b^ (0.835)	18		
*S. aureus* ^B^	Hybrids ^3^	12.92 ^a^ (0.293)	10		
	Lemon	13.23 ^a^ (1.344)	44		
	Lime	14.45 ^a^ (1.673)	9	22	0.002
	Mandarin	13.09 ^a^ (0.942)	12		
	Orange	11.88 ^a^ (1.987)	16		
*Salmonella* ^B^	Lemon	12.77 ^b^ (0.365)	42		
	Lime	16.21 ^a^ (0.256)	7	11	0.086
	Orange	14.59 ^ab^ (1.527)	17		
*L. monocytogenes* ^B^	Lemon	14.56 ^a^ (1.976)	182		
	Mandarin	13.63 ^a^ (1.980)	33	10	0.293
	Orange	13.54 ^a^ (1.977)	152		

^1^ Different superscript uppercase letters mean significant differences in the pooled inhibition diameter produced by the EOs of **lemon and orange** at a dose of 100 mg/mL; A to B: highest to lowest. ^2^ Different superscript lowercase letters mean significant differences in the pooled inhibition diameter against a given bacterium produced by the EOs of *Citrus* species at a dose of 100 mg/mL. ^3^ Category that groups *Citrus medica*, *C. reticulata*, *C. reticulata* cultivar Wilking, *C. japonica* Thunb., and a commercial citrus extract (FOODGARD F410B).

**Table 2:** Pooled inhibition diameters (mean and standard error, SE, in mm) of *Origanum* species extracts against specific bacteria using meta-analysis models. Number of observations (n), number of primary studies (N), and *p*-value of the publication bias test are presented for each model.

Bacterium ^1^	Plant	Method	Pooled Inhibition Diameter ^2^ (SE) (mm)	n	N	Pub. Bias (*p*-Value)
*E. coli* ^C^	Marjoram	Disk	16.58 ^a^ (1.360)	7	18	0.877
	Oregano	Disk and Well ^4^	15.01 ^a^ (1.059)	27		
*B. cereus* ^A^	Oregano	Disk and Well ^4^	22.27 (1.734)	9	6	0.840
*S. aureus* ^AB^	Marjoram	Disk	27.77 ^a^ (2.315)	5		
	Oregano	Disk and Well ^4^	20.15 ^b^ (1.944)	78	20	0.815
	Others ^3^	Well	10.21 ^c^ (0.509)	3		
*Salmonella* ^B^	Greek oregano	Disk	24.68 ^b^ (2.192)	11		
	Marjoram	Disk	19.45 ^a^ (1.079)	22	21	0.130
	Oregano	Disk and Well ^4^	19.29 ^a^ (1.435)	97		
*L. monocytogenes* ^B^	Greek oregano	Disk	44.96 ^a^ (0.297)	10		
	Marjoram	Disk	25.53 ^b^ (0.343)	8		
	Oregano	Disk Well	18.66 ^c^ (1.877) 21.49 ^bc^ (1.015)	45 7	11	0.117
	Others ^3^	Well	13.64 ^d^ (2.699)	6		
STEC ^B^	Greek oregano	Disk	22.71 ^a^ (1.665)	11		
	Marjoram	Disk	15.19 ^b^ (1.885)	14	7	0.348
	Oregano	Disk and Well ^4^	20.05 ^a^ (1.829)	21		

^1^ Different superscript uppercase letters mean significant differences in the pooled inhibition diameter produced by the extracts of **oregano only** at a dose of 100 mg/mL; A to C: highest to lowest. ^2^ Different superscript lowercase letters mean significant differences in the pooled inhibition diameter against a given bacterium produced by extracts of *Origanum* species at a dose of 100 mg/mL. ^3^ Category that groups *Origanum dictamnus*, *O. syriacum*, and *O. minutiflorum.* ^4^ Inhibition diameters from the disk and well method were combined, since the effect of method of determination was not significant (*p* > 0.10).

**Table 3:** Pooled inhibition diameters (mean and standard error, SE, in mm) of *Syzygium aromaticum* extracts against specific bacteria using meta-analysis models. Number of observations (n), number of primary studies (N), and *p*-value of the publication bias test are presented for each model.

Bacterium ^1^	Method	Pooled Inhibition Diameter ^2^ (SE) (mm)	n	N	Pub. Bias (*p*-Value)
*E. coli* ^B^	Disk Well	14.60 ^b^ (0.894) 18.08 ^a^ (1.123)	22 9	14	0.162
*B. cereus* ^A^	Disk Well	16.29 ^b^ (1.399) 20.53 ^a^ (1.359)	15 5	9	0.044
*S. aureus* ^B^	Disk Well	12.86 ^b^ (1.032) 20.10 ^a^ (2.613)	14 9	12	0.293
*Salmonella* ^C^	Disk and Well ^3^	13.17 (1.360)	27	13	0.337
*L. monocytogenes* ^C^	Disk and Well ^3^	15.81 (1.573)	20	12	0.042
STEC ^C^	Disk	12.61 (1.227)	14	4	0.004

^1^ Different superscript uppercase letters mean significant differences in the pooled inhibition diameter produced by extracts of *Syzygium aromaticum* at a dose of 100 mg/mL; A to C: highest to lowest. ^2^ Different superscript lowercase letters mean significant differences in the pooled inhibition diameter against a given bacterium produced by the extracts of *Syzygium aromaticum* at a dose of 100 mg/mL. ^3^ Inhibition diameters from the disk and well method were combined, since the effect of method of determination was not significant (*p* > 0.10).

**Table 4:** Pooled MICs (mean and 95% confidence intervals, CIs) produced by extracts (in mg/mL) or EOs (in μL/mL) of *Citrus* species by method of determination (agar dilution (AD) and broth micro-dilution (BMiD)), as estimated by meta-analysis models separately adjusted by bacterium. Number of observations (n), number of primary studies (N), and *p*-value of the publication bias test are displayed per meta-analysis model.

Bacterium	Plant	Type	Method	MIC ^1^ (95% CI) (mg/mL or μL/mL)	n	N	Pub. Bias (*p*-Value)
*E. coli*	Bitter orange	Extract	AD BMiD	8.692 ^b^ [2.086–36.21] 2.540 ^ab^ [0.877–7.358]	5 6		
	Hybrids	Extract	BMiD	0.841 ^a^ [0.309–2.288]	9	6	0.709
	Lime	Extract	BMiD	3.806 ^ab^ [1.034–14.00]	4		
	Sweet orange	Extract	BMiD	0.283 ^ab^ [0.019–4.342]	6		
*B. cereus*	All ^2^	Extract and EO	BMiD	1.411 [0.527–3.779]	9	4	0.659
*S. aureus*	Bitter orange	Extract	AD BMiD	7.647 ^b^ [1.835–31.86] 2.850 ^b^ [0.984–8.259]	5 6		
	Hybrids	Extract	BMiD	0.552 ^a^ [0.196–1.554]	8		
	Lemon	EO	BMiD	2.365 ^ab^ [0.286–19.56]	3	14	0.283
	Lime	Extract	BMiD	2.298 ^b^ [0.858–6.154]	9		
	Mandarin	EO	BMiD	5.000 ^ab^ [0.308–81.22]	2		
	Sweet orange	Extract	BMiD	0.689 ^ab^ [0.187–2.536]	4		
*Salmonella*	Bitter orange	Extract	AD	10.43 ^b^ [3.505–23.50]	5	4	0.755
	Hybrids	Extract	BMiD	0.796 ^a^ [0.323–1.965]	10		
*L. monocytogenes*	Bitter orange	Extract	AD	8.692 ^b^ [2.086–36.22]	5		
	Hybrids	Extract	BMiD	0.618 ^a^ [0.158–2.410]	4	6	0.946
		EO	BMiD	2.500 ^ab^ [0.995–6.281]	3		
	Lemon	EO	AD	0.884 ^ab^ [0.179–4.358]	4		

^1^ Within a given bacterium, where a meta-analysis model was fitted, different superscript lowercase letters mean significant differences in MIC produced by extracts and EOs of *Citrus* species. ^2^ No significant differences were found between *Citrus* species.

**Table 5:** Pooled MICs (mean and 95% confidence intervals, Cis) produced by extracts (in mg/mL) or EOs (in μL/mL) of *Origanum* species by method of determination (agar dilution (AD), broth macro-dilution (BmaD) and broth micro-dilution (BmiD)), as estimated by meta-analysis models separately adjusted by bacterium. Number of observations (n), number of primary studies (N), and *p*-value of the publication bias test are displayed per meta-analysis model.

Bacterium	Plant	Type	Method	MIC ^1^ (95% CI) (mg/mL or μL/mL)	n	N	Pub. Bias (*p*-Value)
*E. coli*	Marjoram	Extract	BmiD	3.876 ^b^ [0.573–26.22]	5	30	0.172
	Oregano	Extract	All ^2^	0.566 ^ab^ [0.197–1.629]	39		
		EO	BmiD	0.018 ^a^ [0.001–0.437]	12		
*B. cereus*	Oregano	Extract	BmiD	1.664 ^a^ [0.412–6.719]	8	9	0.021
		EO	BmiD	3.681 ^a^ [0.610–22.22]	4		
*S. aureus*	Marjoram	Extract	BmiD	2.219 ^c^ [1.843–2.670]	103	42	0.749
	Oregano	Extract	AD BmaD BmiD	1.013 ^b^ [0.467–2.196] 0.098 ^a^ [0.035–0.276] 0.389 ^b^ [0.255–0.593]	17 9 44		
		EO	BmaD BmiD	1.053 ^bc^ [0.172–6.459] 1.219 ^c^ [0.557–2.665]	5 56		
	Za’atar	EO	BmiD	0.363 ^ab^ [0.057–2.313]	4		
*Salmonella*	Marjoram	Extract	BmiD	2.161 ^b^ [0.519–9.003]	4	26	0.075
	Oregano	Extract	BmiD	0.473 ^a^ [0.192–1.168]	32		
		EO	BmiD	1.319 ^b^ [0.671–2.594]	56		
*L. monocytogenes*	Marjoram	EO	BmiD	1.901 ^b^ [0.256–14.12]	3	22	0.850
	Oregano	Extract	BmaD BmiD	0.129 ^a^ [0.042–0.401] 0.558 ^b^ [0.242–1.293]	8 9		
		EO	BmaD BmiD	0.822 ^b^ [0.209–3.229] 1.204 ^b^ [0.723–2.006]	3 60		
STEC	Oregano	Extract	BmiD	0.394 ^a^ [0.107–1.448]	4	5	0.554
		EO	BmiD	0.364 ^a^ [0.139–0.953]	5		

^1^ Within a given bacterium, where a meta-analysis model was fitted, different superscript lowercase letters mean significant differences (*p* < 0.10) in MIC produced by extracts and EOs of *Origanum* species. ^2^ MICs measured by AD, BmaD, and BmiD were combined since the effect of method of determination was not significant (*p* > 0.10).

**Table 6:** Pooled MICs (mean and 95% confidence intervals, CIs) produced by extracts (in mg/mL) or EOs (in μL/mL) of clove by method of determination (agar dilution (AD) and broth micro-dilution (BMiD)), as estimated by meta-analysis models separately adjusted by bacterium. Number of observations (n), number of primary studies (N), and *p*-value of the publication bias test are displayed per meta-analysis model.

Bacterium	Type	Method	MIC ^1^ (95% CI) (mg/mL or μL/mL)	n	N	Pub. Bias (*p*-Value)
*E. coli*	Extract and EO	AD and BMiD ^2^	0.080 [0.004–1.837]	11	8	0.970
*B. cereus*	Extract	AD and BMiD ^2^	4.978 [1.552–15.96]	5	4	ND ^3^
*S. aureus*	Extract	AD and BMiD ^2^	0.313 ^a^ [0.028–3.519]	11	7	ND ^3^
	EO	BMiD	1.047 ^a^ [0.166–6.606]	3		
*Salmonella*	Extract	BMiD	0.815 ^a^ [0.358–1.858]	9	8	0.298
	EO	BMiD	1.854 ^a^ [0.620–5.540]	6		
*L. monocytogenes*	EO	BMiD	1.029 [0.417–2.539]	8	5	0.877

^1^ Within a given combination plant × bacterium, where a meta-analysis model was fitted, different superscript lowercase letters mean significant differences in MIC against a given bacterium produced by extracts and EOs. ^2^ MIC from AD and BMiD were combined, since the effect of method of determination was not significant (*p* > 0.10). ^3^ Effect of study size could not be determined since it was the same across all outcomes (*p* > 0.10).

**Table 7:** Meta-regression analysis of the inhibitory diameter induced by extracts from *Origanum* (*n* = 145), *Syzygium aromaticum* (*n* = 10), and *Citrus* (*n* = 7) plants, as a function of the MIC (mg/mL for extracts and μL/mL for EOs), extract dose (%), and bacterium. Number of observations (n) per factor level, heterogeneity analysis, and *p*-value of the publication bias test are presented.

Parameter	Estimate ^1^	Standard Error	*p*-Value	n	Heterogeneity Analysis ^2^
Intercept	−1.515	6.499	0.816		
Log MIC	−5.554	0.181	<0.0001		s^2^ = 29.34
Log Dose	18.00	0.227	<0.0001		τ^2^ = 33.96
Bacterium					I^2^ = 53.6%
*L. monocytogenes*	1.319 ^b^	0.150	<0.0001	43	τ^2^_res_ = 17.75
*S. aureus*	2.668 ^c^	0.146	<0.0001	37	R^2^ = 47.7%
*Salmonella*	2.429 ^c^	0.141	<0.0001	41	
STEC	−0.411 ^a^	0.234	<0.0001	9	Publication bias
*C. jejuni*	-	-	-	32	*p* = 0.254

^1^ Superscript letters indicate significant differences in the estimates among bacteria. ^2^ The heterogeneity analysis comprises the following components: within-study variability (s^2^), between-study variability of the null model (τ^2^), intra-class correlation (I^2^), residual between-study variability (τ^2^_res_), and between-study variability explained by significant moderators (R^2^).

## Data Availability

Summary data are available upon request.

## Supplementary Materials

The following supporting information can be downloaded at: https://www.mdpi.com/article/10.3390/foods12061265/s1, Figure S1: Funnel plots of meta-analysis models on inhibition diameters produced by essential oils of *Citrus* species for each bacterium. Markers symbolise species: ○ = hybrids, ∆ = lemon, + = lime, × = mandarin, ◊ = orange; Figure S2: Funnel plots of meta-analysis models on inhibition diameters produced by extracts of *Origanum* species for each bacterium. Markers symbolise species: □ = Greek oregano, ○ = marjoram, ∆ = oregano, + = others; Figure S3: Funnel plots of meta-analysis models on inhibition diameters produced by extracts of *Syzygium aromaticum* for each bacterium. Markers symbolise method of determination: ○ = disk, ∆ = well; Figure S4: Funnel plots of meta-analysis models on MICs of extracts/EOs of clove for each bacterium. Markers symbolise method of determination: □ = agar dilution, ∆ = broth micro-dilution; Figure S5: Funnel plots of meta-analysis models on MICs of extracts/EOs of *Citrus* species for each bacterium. Markers symbolise plant species: □ = bitter orange, ○ = hybrids, ∆ = lemon, + = lime, × = mandarin, ◊ = sweet orange; Figure S6: Funnel plots of meta-analysis models on MICs of extracts/EOs of *Origanum* species for each bacterium. Markers symbolise species: □ = marjoram, ○ = oregano, ∆ = zaatar; Figure S7: Funnel plot of the meta-regression model on inhibition diameters produced by extracts of *Origanum* (*n* = 145), *Syzygium aromaticum* (*n* = 10), and *Citrus* (*n* = 7) plants. Markers symbolise bacteria: □ = *C. jejuni*, ○ = *L. monocytogenes*, ∆= *S. aureus*, + = *Salmonella*, × = STEC.
