# Supplementary material for: Meta-Analysis of In Vitro Antimicrobial Capacity of Extracts and Essential Oils of Syzygium aromaticum, Citrus L. and Origanum L.: Contrasting the Results of Different Antimicrobial Susceptibility Methods
Source: Foods. 2023 Mar 16;12(6):1265. doi: 10.3390/foods12061265 (PMC10048651; doi:10.3390/foods12061265)
Supplement: Supplementary file 1 [file foods-12-01265-s001.zip › foods-2268762-supplementary.pdf]

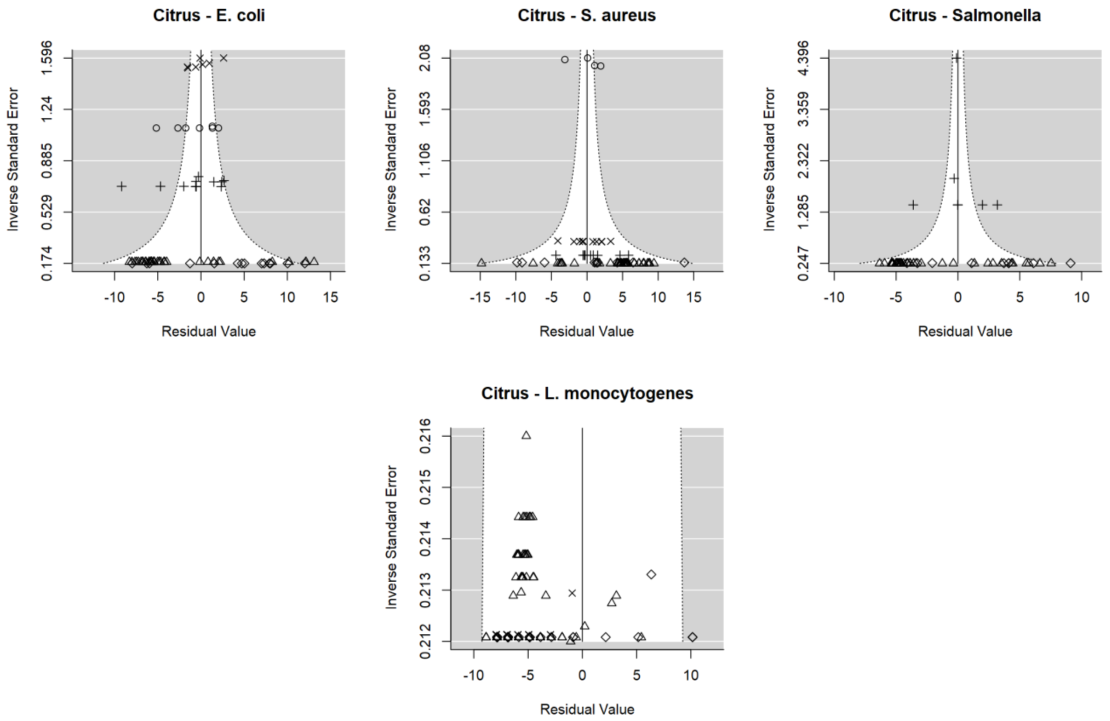

Figure S1. Funnel plots of meta-analysis models on inhibition diameters produced by essential oils of *Citrus* species for each bacterium. Markers symbolise species: ○=Hybrids, Δ=Lemon,+=Lime,×=Mandarin, ◊=Orange

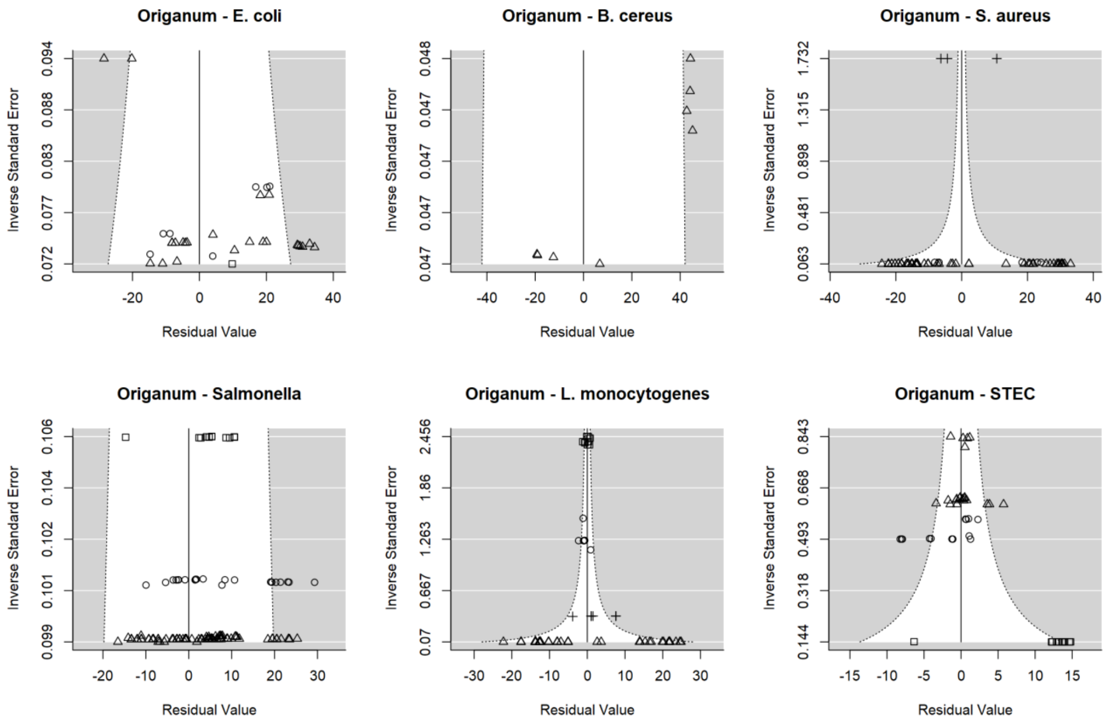

Figure S2. Funnel plots of meta-analysis models on inhibition diameters produced by extracts of *Origanum* species for each bacterium. Markers symbolise species: □=Greek oregano, ○=Marjoram, Δ=Oregano, +=Others

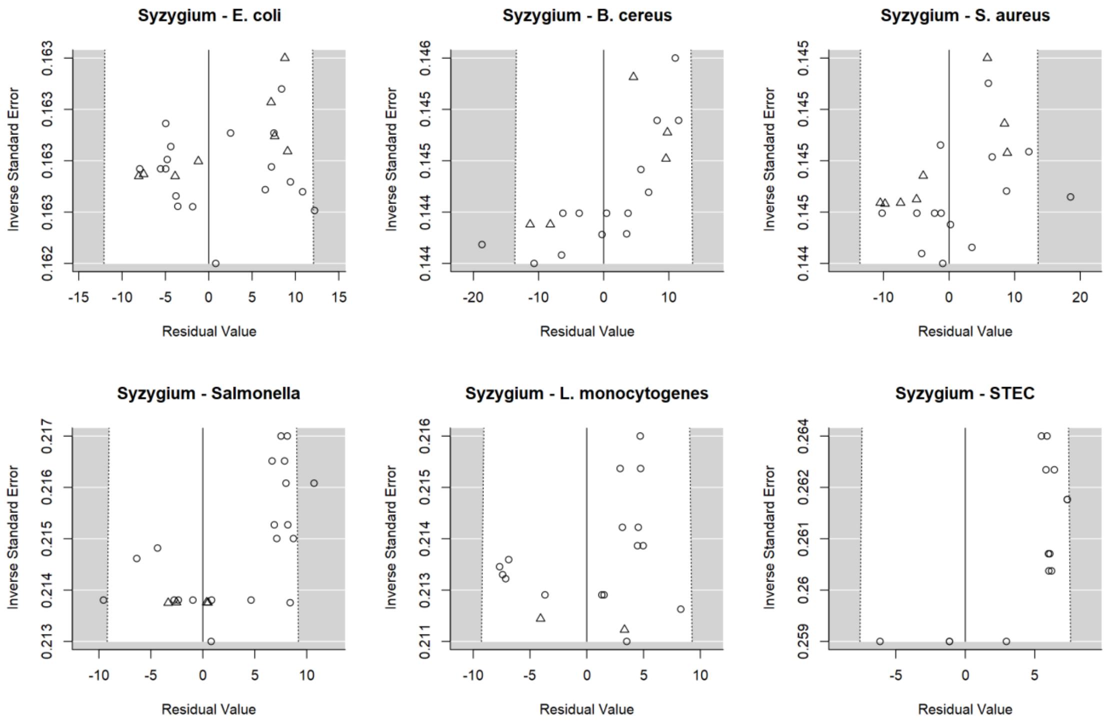

Figure S3. Funnel plots of meta-analysis models on inhibition diameters produced by extracts of *Syzygium aromaticum* for each bacterium. Markers symbolise method of determination: ○=Disk, Δ=Well

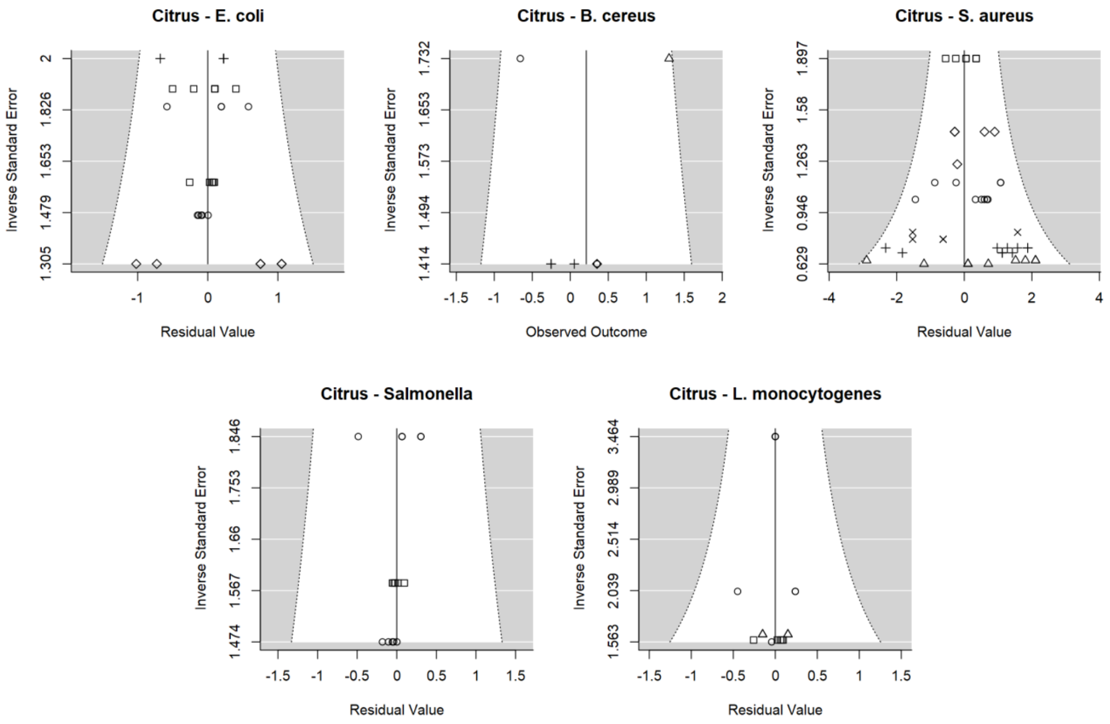

Figure S4. Funnel plots of meta-analysis models on MICs of extracts/EOs of *Citrus* species for each bacterium. Markers symbolise plant species: □=Bitter orange, ○=Hybrids, Δ=Lemon, +=Lime, ×=Mandarin, ◊=Sweet orange

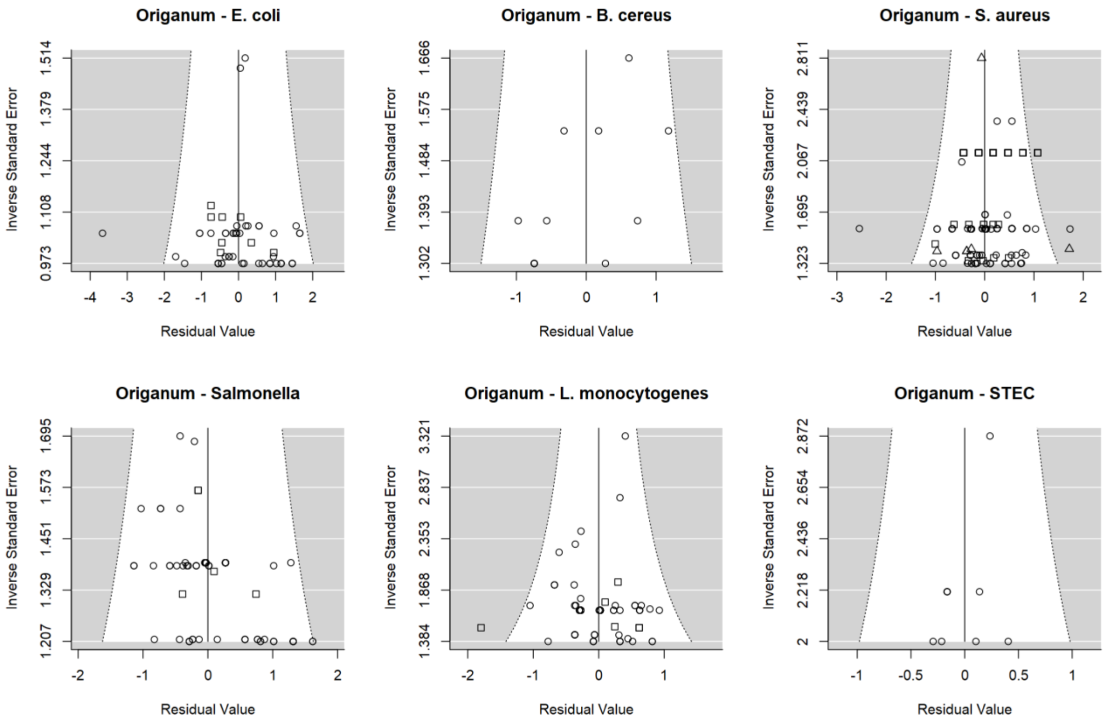

Figure S5. Funnel plots of meta-analysis models on MICs of extracts/EOs of *Origanum* species for each bacterium. Markers symbolise species: □=Marjoram, ○=Oregano, Δ=Zaatar

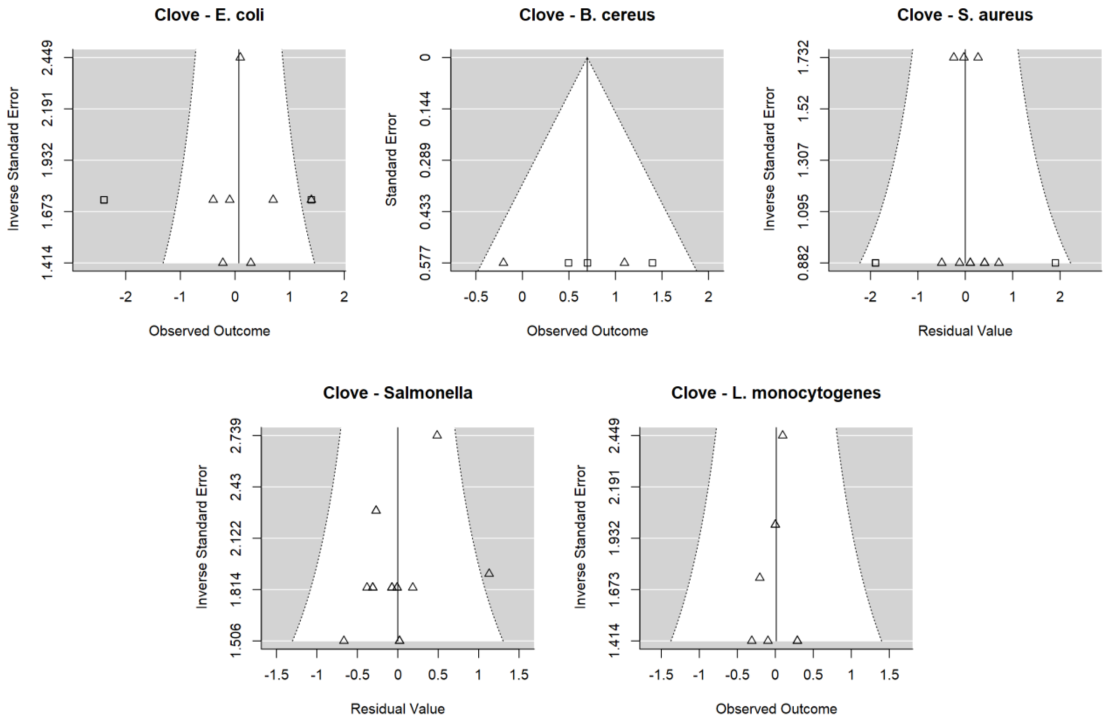

Figure S6. Funnel plots of meta-analysis models on MICs of extracts/EOs of clove for each bacterium. Markers symbolise method of determination: □=Agar dilution, Δ=Broth micro-dilution

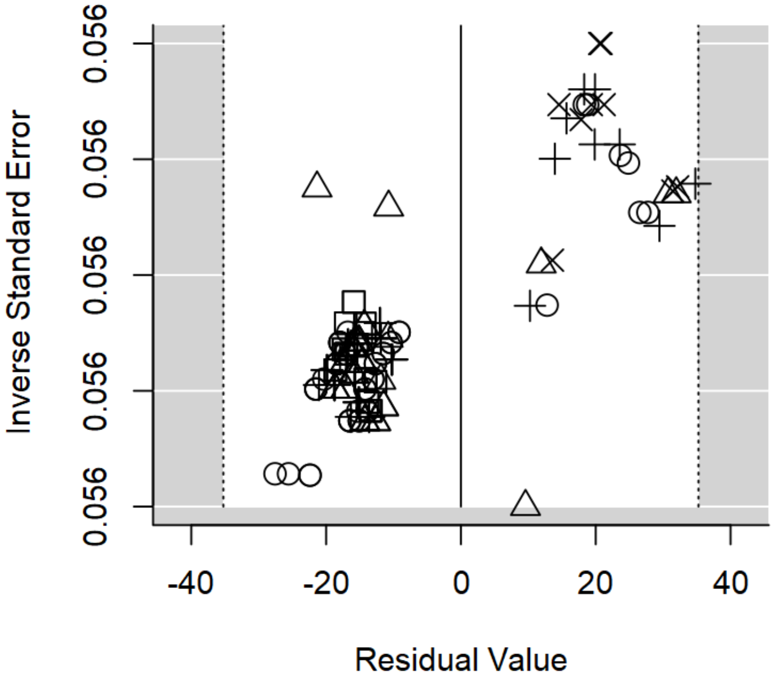

Figure S7. Funnel plot of the meta-regression model on inhibition diameters produced by extracts of *Origanum* (n=145), *Syzygium aromaticum* (n=10) and *Citrus* (n=7) plants. Markers symbolise bacterium: □= *C. jejuni*, ○= *L. monocytogenes*, Δ= *S. aureus*, += *Salmonella*, ×= STEC
